# Ascertaining Susceptibilities in Smart Contracts: A Quantum Machine Learning Approach

**DOI:** 10.3390/e27090933

**Published:** 2025-09-04

**Authors:** Amulyashree Sridhar, Kalyan Nagaraj, Shambhavi Bangalore Ravi, Sindhu Kurup

**Affiliations:** 1 Department of Computer Science and Engineering, Amrita School of Computing, Amrita Vishwa Vidyapeetham, Bengaluru Campus, Bengaluru 560035, Karnataka, India; s_amulyashree@blr.amrita.edu; 2 Department of Computer Science and Engineering (Data Science), B.M.S. College of Engineering, Basavanagudi, Bangalore 560019, Karnataka, India; shambhavibr.ise@bmsce.ac.in (S.B.R.); ksindhu.ise@bmsce.ac.in (S.K.)

**Keywords:** smart contracts, liabilities, QML, QNN, McNemar’s Test

## Abstract

The current research aims to discover applications of QML approaches in realizing liabilities within smart contracts. These contracts are essential commodities of the blockchain interface and are also decisive in developing decentralized products. But liabilities in smart contracts could result in unfamiliar system failures. Presently, static detection tools are utilized to discover accountabilities. However, they could result in instances of false narratives due to their dependency on predefined rules. In addition, these policies can often be superseded, failing to generalize on new contracts. The detection of liabilities with ML approaches, correspondingly, has certain limitations with contract size due to storage and performance issues. Nevertheless, employing QML approaches could be beneficial as they do not necessitate any preconceived rules. They often learn from data attributes during the training process and are employed as alternatives to ML approaches in terms of storage and performance. The present study employs four QML approaches, namely, QNN, QSVM, VQC, and QRF, for discovering susceptibilities. Experimentation revealed that the QNN model surpasses other approaches in detecting liabilities, with a performance accuracy of 82.43%. To further validate its feasibility and performance, the model was assessed on a several-partition test dataset, i.e., SolidiFI data, and the outcomes remained consistent. Additionally, the performance of the model was statistically validated using McNemar’s test.

## 1. Introduction

Centralized systems were proposed to facilitate parties performing commercial transactions by connecting with each other using a trustworthy third-party dealer (like banks). However, entrusting third-party vendors has often proceeded to infringement of privacy and security inconsistencies, also leading to towering transactional overheads [1]. Blockchain technology was proposed to overcome these disputes by permitting contributors to reach an agreement regarding their transactions without any association with a reliable third party [2]. Blockchain can be understood as a distributed databank that preserves the history of all negotiations that have happened within its network [3,4]. It is also the revolutionary method behind the first distributed automated payment system, i.e., Bitcoin [5].

Blockchain has further progressed to assist in abundant decentralized approaches which are beyond the scope of economic operations. These implementations depend on the performance of smart contracts within the blockchain network. Smart contracts were conceptualized by Szabo [6]; they are basically computer programs which decode a covenant, in the form of a digital contract, based on predefined, concise rules. These contracts are deployed within the blockchain network for executing transactions, designed to replace traditional paper-based agreements [7]. Smart-contract-facilitated transactions are ideally transparent, unalterable, and distinguishable. These contracts are usually transcribed in explicit domain-based languages, including Solidity, Pact, and Liquidity, for the Ethereum, Kadena and Tezos platforms. These contracts are deployed and executed by miners, which are a special type of participants employed for executing transactions in the blockchain. Miners are paid for their job based on the computational overheads required for completing a task [8]. With the progression of smart contracts, enormous numbers of contracts are being deployed presently on the blockchain, making it impracticable to employ manual examination [9]. There are strong possibilities of security breaches as the contract layer within the blockchain network has pronounced involvement with the blockchain mechanism, often leading to storage and resource exploitation [10]. Initially, a miner node is identified to create a block within the network. However, the location of the miner node is habitually unverified, whether it is being executed from a trusted environment or not. Suppose the miner node turns out to be malevolent. It could then manipulate complete transactions within the block, resulting in major security breaches [11]. Also, contracts need to call one another iteratively to attain complicated functionality. However, referring to an external duplicitous contract could raise the possibility of errors and security threats if the contract turned out to be vindictive [12]. Also, as smart contracts can be inscribed and deployed by any user with various coding capabilities and designing platforms, there is no assurance for anyone deploying smart contracts without security constraints [13]. Similarly, smart contracts have exclusive features, like the gas mechanism, as compared to conventional codes. Suppose a smart contract is poorly designed. It could then utilize much of the gas, resulting in a halting of operations due to excessive resource consumption [14,15]. Correspondingly, due to the remarkable relationship between smart contracts and financial markets, numerous attacks have been executed by manipulating susceptibilities of smart contracts for earning profit deployment [16]. To ensure data reliability and distinguishability of transactions, contracts, once executed, are unable to be altered post-deployment.

Susceptibility in contracts refers to any code-level weakness, design flaw, or operational condition that could potentially be exploited to cause undesired behavior, resource exhaustion, or financial loss. Detecting these vulnerabilities is one of the essential aspects of the development and disposition of smart contracts. In cases when susceptibilities are identified, they cannot be mitigated by upgrading versions or patching due to evidence of tampering; hence, they must be often self-destructed. Therefore, it is evident that the presence of malignant attacks cannot be prevented on smart contracts. An instance of this is the famous “DAO (Decentralized Autonomous Organization)” incident, which accounted for about ETH 2 million (i.e., USD 50 million) in financial losses due to the exploitation of reentrancy vulnerability by attackers. Reentrant issues led to susceptibilities in the contract, which were exploited by a recursive call to the “splitDAO” function within the contract to withdraw ETH [17]. It is evident that gaining control of smart contracts has resulted in unbearable losses, for example, by manipulating the bZx protocol, where virtual assets were withdrawn by attackers by restraining the oracle, ensuring a profit of ETH 2000 or more [18]. These attacks reflect the impact on financial assets in cases when smart contracts are attacked and manipulated. Therefore, it is vital to detect liabilities in contracts at an earlier stage to mitigate security breaches of assets. Detecting vulnerabilities is one of the essential aspects in the development and disposition of smart contracts [19].

Currently, susceptibilities in smart contracts are identified using numerous approaches including human evaluation [20], fuzz analysis [21], static investigation [22] and precise authentication. Also, vulnerability detection tools like Mythril [23], Oyente [24], Slither [25], Securify [26] and Smartcheck [27] have been adopted to ascertain attacks on smart contracts. These tools interpret the code within contracts to recognize diverse vulnerabilities, including relinquished exceptions, timestamp reliance, reentrancy and improper tx.origin consent. However, these tools sometimes generate false negatives and false positives instances as they are extremely dependent on preconceived recognition rules and lack the competence to precisely capture composite logic. Also, these premediated rules become obsolete over a point of time, making it challenging to acclimatize and produce new data, which is essential for analyzing large instances of smart contracts in less amount of time [28].

Moreover, detecting unknown vulnerabilities in smart contracts is a challenging issue in smart contracts [29]. It is worth noting that machine learning (ML) algorithms have been adopted for detecting these vulnerabilities, as they are known to capture liabilities by apprehending knowledge patterns in less time with improved performance [30]. Previous studies have shown that employing ML models to detect obligations in smart contracts has led to substantial amendments in vulnerability detection as compared to traditional vulnerability detection techniques [31,32]. Specifically, deep learning (DL) technology has been adopted ardently as an effective approach to detect vulnerabilities within contracts. These models are employed to extract relevant attributes from large data to detect liabilities [33]. Diverse variants of DL techniques have been employed to detect vulnerabilities [34,35,36,37]. However, some DL approaches habitually disregard implication of liable attributes during data pre-processing tasks, while some models lack competence in semantic examination, leading to instances of false positives in identifying vulnerabilities [38,39,40]. Despite its widespread application in vulnerability detection, ML algorithms have certain limitations. Additionally, as the size of contracts upsurges, the cost of training ML algorithms also intensifies exponentially. Furthermore, expansion of classical ML algorithms would also result in storage and performance constraints [41]. All these encounters must be addressed while employing ML approaches in detecting smart contract vulnerabilities.

Quantum computing, on the other hand, can be used to solve storage constraints of classical computers. The field is centered on fundamentals of quantum mechanics, including qubits (i.e., quantum bits), superposition, entanglement and interference, for processing data. As compared to a classical bit, qubit could either have a zero state, one state or an amalgamation of two states at an equivalent time, which is recognized as linear superposition [42,43]. Quantum computers also employ ML approaches to solve concrete glitches with improved accuracy and speed, as compared to classical computers. The domain that combines quantum computers with ML algorithms is referred to as ‘Quantum Machine Learning’ (QML) [44,45]. At present, QML approaches have been employed in fields of natural language processing (NLP) [46,47,48], speech recognition [49], recommendation systems [50] and image processing [51]. These QML techniques are known to enhance the execution time and efficiency of programs. They are also recognized to perform better than traditional DL algorithms through effective training and learning from large data [52,53].

Centered on above fundamentals, the current study attempts to detect liabilities in smart contracts by employing QML algorithms [54]. To the best of our awareness, there has been very limited research attempted to identify and validate smart contract vulnerabilities by employing QML approaches [55]. These techniques are known to solve memory and performance constraints of traditional ML algorithms. Feasibility of these techniques is examined towards uncovering liabilities in smart contracts. The contributions of the present study are summarized below:

- Presently, no research has been attempted to detect smart contract vulnerabilities using QML algorithms. To fill this existing gap, the current study employs four well known QML algorithms to detect vulnerabilities.
- Compared to classical ML algorithms, QML algorithms were able to process knowledge within smart contracts by consuming less memory and by utilizing the benefits of quantum mechanics. In addition to liability detection, the current work also implies prospects of network security implications from outcomes of QML approaches.
- Employing QML models for detecting liabilities in smart contracts on a larger scale would be beneficial. Non-uniform lengths of contracts were analyzed effectively using QML algorithms, especially by the quantum neural network (QNN) approach, thus solving the problem of inconsistent input lengths. Also, vulnerabilities were detected in batches using these QML algorithms, reflecting the capability of batch processing.
- Experimentations revealed that the QNN model outperformed other QML algorithms in detecting vulnerabilities with a performance accuracy of 81.78%. This accuracy was further improved to 97.63% on a test dataset. The average accuracy of the model on small-scale smart contracts datasets derived from splitting the test dataset was found to be 89.96%.

## 2. Literature Survey

This section highlights significance of previous studies in discovering vulnerabilities from smart contracts.

### 2.1. Significance of Security in Smart Contracts

Smart contracts are a variant of digital arrangement, which automatically operates on a blockchain network, given gratification of explicit conditions. They are programmatically similar to ‘If-then’ statements, which could be executed without the need of a third party (i.e., decentralized architecture). These contracts include code attributes for storing details, distributing ethers and collaborating with other contracts that aid in decision making. They empower reliable transactions, which are often irretrievable and perceptible [56]. Compared to conventional contracts, smart contracts are often revered for their effectiveness in minimizing transactional risks, service and administrative costs, which in turn improves the efficacy of business transactions [57]. As of January 2022, over 637 million smart contracts have been deployed in Ethereum Virtual Machines (EVMs) [58].

Despite numerous benefits, these contracts have certain limitations which need to be addressed. Regardless of being an asset in blockchain [59], they are often targeted by hackers. The reason is quite simple: these contracts frequently store excessive amounts of implicit currency and execute immutable code based on previously deployed contracts, often resulting in disposed fortification, which could be exploited [60]. Also, these contracts are immutable; once deployed, any security issues cannot be tackled, leading to an irretrievable dent. Recently, innumerable security breaches have arisen while developing and deploying these contracts. All these mishaps have resulted in extensive financial losses. One such instance is the attack that happened on Binance (BNB) smart chain (i.e., BSC), which is a platform for developing high-performance decentralized blockchain applications. On 7 October 2022, BNB chain was attacked by hackers to loot about two million coins within a span of two hours. The attack was targeted towards BSC token core, leading to issuance of surplus BNB [61].

It can be concluded that despite value-added benefits from smart contracts, vulnerabilities in privacy do exist to date. Even an insignificant violation of security could lead to colossal losses. These obligations in contracts also dispute the grounds of entrust in blockchain networks. As these contracts are still advancing, they are not yet accurate; security assurance has gathered substantial interest among researchers.

### 2.2. Conventional Static Techniques

Numerous tools are currently available to detect obligations in smart contracts. Some popular approaches are discussed below.

Mythril (i.e., MythX) safety analysis approach was designed to examine bytecode from contracts that were executed in EVM. In case liabilities were discovered, it would aid in investigating probable sources by exploring input data [62]. This tool was beneficial in uncovering active exposures, hence curtailing the prospect of exploitation. Primarily, symbolic examination and taint analysis were incorporated to detect liabilities. Nevertheless, while executing taint analysis, restrictions were recognized due to memory constraints. This hindrance would increase in intensity while handling reference-style attribute requests. Furthermore, Mythril would also confront state explosion issues while administering multifaceted smart contracts. Correspondingly, symbolic execution employed to identify liabilities might take branches into consideration, which are not feasible in reality, often resulting in instances of false positives.

Smartcheck is another vulnerability detection approach employed on smart contracts [27]. It is centered on statistical procedures that generate a quintet of rule sets to discover liabilities in contracts. With reliance on logic rules, there are likelihoods of generating false negatives and false positives instances in detecting obligations. Similarly, it may breakdown sometimes while recognizing dangerous programming errors, resulting in imprecise inferences from overseen liabilities.

As another tool, Slither [25] is used to identify liabilities and probable glitches in Solidity platform smart contracts. It combines abundant detectors capable of discovering diverse liabilities. As compared to Mythril, it executes faster and effectively detects vulnerabilities. But it requires conventional semantic analysis that confines its capability to perform thorough security investigation to identify inferior level details like gas computations.

### 2.3. ML Approaches Towards Vulnerability Detection

ML is a subdiscipline of Artificial Intelligence (AI), which supports computers to study prevalent data to discover patterns that aid in decisions and predictions without being obviously programmed [63]. A study [64] discovered 16 diverse vulnerable patterns within contracts in the Solidity platform using ML approaches with a performance accuracy of 95%. But the study was centered on static code analysis on a small sample of about 1000 smart contracts, limiting its scalability. Another research study highlighted the deficiencies of traditional susceptibility detection techniques and discussed the possibility of employing ML approaches to discover liabilities in contracts [65]. However, the study does not analyze these liabilities within contracts and propose solutions. Yet another study identified three variants of obligations in smart contracts by employing multi-task learning approaches, centered on hard attribute sharing [66]. When contrasted with single-task learning, this model executed better in terms of storage, time and computation. A multi objective neural network model (MODNN) was proposed in another study [67] to ascertain liabilities in smart contracts. The model discovered about 10 recognizable menaces along with unknown types of threats. Vulnerabilities were discovered in parallel mode with an average performance of 94.8% F1 score, depicting better scalability. However, this model could not capture raw semantic and syntactic details from contracts. Another study employed the DL model to detect five types of susceptibilities in smart contracts by considering control-flow modes, source code details and operation code features. The model achieved enhanced performance in terms of accuracy and Area Under the Curve (AUC) parameters [68]. A framework, MANDO-HGT, was proposed [69] to discover vulnerabilities in smart contracts. This framework analyzed about 55,000 Ethereum contracts to generate heterogeneous contact graphs (HCGs) that identify obligations. Experimentations reflected the performance of this framework on contracts with an F1 score of 0.7%. Another study identified liabilities in smart contracts based on opcodes. Here, a control-flow graph (CFG) was used for training a novel Multi-layer Perceptron (MLP) model designed from multiple benchmark ML models. The model identified obligations with an accuracy of 91% and false positive rate (FPR) of 0.0125. However, the exact location of source bugs could not be traced using this approach [70]. Ensemble models [71] and improvisations in DL approaches [72] are also proposed to detect liabilities.

However, these approaches sometimes disregard the dynamics of smart contracts as they process them as a sequence of text, by ignoring the data flow and logic part [73].

The above-mentioned approaches have been extensively employed to ascertain liabilities in smart contracts. But these methods ignore certain crucial susceptible attributes during preprocessing [74]. Similarly, some of these techniques may have unsatisfactory understanding of semantics of liable programs, often resulting in instances of false positives [75].

To resolve these issues, the current study employed feature processing followed by liability detection. The Principal Component Analysis (PCA) algorithm [76] was employed for tumbling dimensionality of features in smart contracts, by maintaining variance amongst them. The features detected were further encoded to QML algorithms for detecting liabilities. Correspondingly, four popular QML algorithms, namely, QNN, variational quantum classifier (VQC), Quantum Support Vector Machine (QSVM) and Quantum Random Forest (QRF), were employed on smart contracts to discover liabilities. Additionally, the performance of these algorithms was evaluated for diverse forms of vulnerabilities in trained contracts.

### 2.4. Recent Advances

Over the years, the domain of security in smart contract has grown significantly, moving beyond rule-based static analysis to incorporate progressive learning approaches, which exploit the rich structural and semantic details. A prominent direction has been graph-based susceptibility detection, where heterogeneous graphs identify numerous associations within contract code, like data-flow, control-flow, and function-call dependencies. These approaches leverage graph neural networks (GNNs) or embeddings to capture both local and global context, thereby improving detection and localization of susceptibilities.

A study [77] proposed an imbalanced heterogeneous graph embedding model for detecting malware on skewed datasets, offering valuable insights for security tasks with rare positive cases in context of smart contracts. Similarly, MANDO and MANDO-GURU were introduced in another study [78], which are multi-level heterogeneous graph embedding models, capable of localization of susceptibilities, via hierarchical structure learning.

Another trend involved multi-view and augmented representations, where different code perspectives, like abstract syntax trees, opcode sequences and control-flow graphs, were fused to derive complementary statistics. Recent systematic reviews on AI-based vulnerability detection using DL approaches have emphasized the use of richer representations to improve performance accuracy and interpretability, which might be computationally expensive [79].

Based on these fundamentals and disadvantages of static approaches and classical ML models in detecting vulnerabilities, the current study proposes a QML framework for detecting smart contract susceptibilities. The study aims to extract opcode-level features from real-time smart contracts and reduces its dimensionality via PCA for effective representation. Further, the study intends to apply and compare multiple QML algorithms, QNN, QSVM, VQC and QRF, to assess their ability in detecting and categorizing contract vulnerabilities. Also, the study intends to evaluate performance of these quantum models against baseline approaches to determine probable improvements in accuracy, false positive, true positive rates and computational efficiency of vulnerability detection.

## 3. Methodology

**Figure 1:**
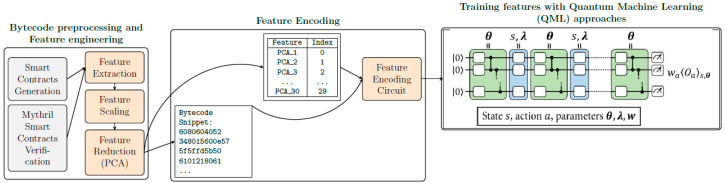
Protocol for identifying susceptibilities in smart contracts.

The following section illustrates methodology of present study in detecting vulnerabilities in smart contracts. The smart contract datasets are initially pre-processed to map with corresponding liabilities. Further, these datasets are deployed on the Solidity platform to extract bytecodes. Additionally, generated bytecodes were validated using the Mythril tool, to avoid discrepancies. The bytecodes generated were further modeled to extract opcodes. Moreover, the opcodes generated a 258-dimension feature set, which was further subjected to feature reduction. Finally, the reduced features were employed to design the quantum circuit for implementing QML algorithms on datasets. The performance of these models in detecting susceptibilities was validated using McNemar’s test. The protocol of current study is visualized as Figure 1.

### 3.1. Notation and Assumptions

Table 1 presents all mathematical symbols employed in the study, along with their denotation and dimensions.

### 3.2. Data Collection and Preprocessing

For identifying susceptibilities, the present study gathered three datasets, namely, Slither smart contract audited dataset [80], wild smartbugs dataset [81] and expert audited smart contract dataset. The Slither dataset included 10,000 smart contracts, while wild smartbugs dataset included 20,000 smart contracts. Also, expert audited dataset included 5000 smart contracts with susceptibilities. To effectively evaluate consequences from auditing tools, the SolidiFI dataset was used as the benchmark test dataset. This dataset included instances of smart contracts with 9369 bugs. The dataset also includes three static vulnerability detection tools, like, Smartcheck, Mythril and Slither to identify five instances of smart contract susceptibilities including Denial of Service (DoS) attacks (i.e., gaslimit), uninitialized storage pointers, access control, reentrancy attacks and overflow. To ensure fair-mindedness and totality of conclusions, QML models were centered on these five types of susceptibilities.

As the entire smart contract could encompass numerous Solidity documents, with a sole Solidity document containing multiple susceptible code snippets, the Slither platform was used to collect 30,000 codes including five instances of liabilities. Moreover, 5000 code snippets audited by experts were manually annotated to identify susceptibilities. Thereby, the training set included 35,000 code fragments. Similarly, for the test dataset, 5000 code fragments were mapped to five instances of vulnerabilities. Description of datasets utilized in study are reflected in Table 2.

### 3.3. Data Cleaning and Splitting

The 35,000 code fragments from smart contracts were further subjected to a cleaning process to eliminate repetitive, redundant, unoccupied and unacceptable instances. Moreover, smart contracts were deployed on a blockchain network using Solidity programming language. Also, code fragments within contracts were compiled to generate bytecodes. Bytecodes are bytes of arrays which are encoded as hexadecimal numerals. They represent explicit sequences of procedures and constraints. Nonetheless, they are known to require huge volumes of memory space to construe and model long arrangements [82]. Therefore, it is impracticable to employ bytecodes reliably as input to QML approaches. Furthermore, the bytecodes generated were validated by the Mythril tool, to circumvent false positive instances. The bytecode representation is also known to have a strong association with opcode in blockchain; therefore, it is possible to discover susceptibilities within contracts with the help of these opcodes. In this perspective, it is essential to convert bytecode to opcode. Opcodes include multiple features and instructions; they can be abridged by mining pertinent features and categorizing them [83].

### 3.4. Opcode Processing and Feature Extraction

Each opcode from smart contracts comprises a 258-dimensional feature set. But processing all these features would pose a dimensional difficulty. Hence, features within opcodes were generalized to create an optimal subset of attributes with the same variability as actual features. In this context, attribute selection approaches like PCA, variance threshold and mutual information (MI) were employed to generate the optimal attribute subset.

### 3.5. Quantum Encoding and Circuit Design

Attributes selected from feature reduction approaches are further subjected to a normalization procedure to convert them into binary representations. Further, the encoded features were fitted into a quantum circuit, based on quantum state preparation. Here, preparation comprises priming quantum registers to train features. This step must be performed effectually to augment potential advantages of QML algorithms. However, initializing quantum states may result in expensive computations with respect to depth and width of quantum circuit [84].

In this context, diverse approaches are proposed in the literature to encode data features to a quantum state. The current study encodes binary data from normalized features to deduce a quantum state |S⟩ that depicts the dataset of *F* features each of *G* instances with length *k* bits each, across linear time in *k* and *F*. Further, the quantum circuit is designed based on the encoding approach adopted in this study [85]. The circuit required +2 qubits (i.e., basic unit to encode data in quantum computing), to encode ***G*** binary instances, which were logically organized as follows:

- A *k*-qubit register $|a^{b}\rangle =|a_{0}^{b}\;a_{1}^{b}\cdots a_{k-1}^{b}\rangle$ for *G* binary instances.
- A 2-qubit ancillary register $|c\rangle$.

The register |*ab*⟩ included qubits as instance of bits which were intended for encoding. Thereby, a binary input could be characterized by reversing qubits from 0 to 1 based on the instances of input bits. The steps for encoding the feature instance $\;{\left(b+1\right)}^{th\;a^{\left(b\right)}}$, presuming previous *b* instances were encoded, are outlined below:

- Selectively reverse $|a^{b}\rangle \;$ qubits for matching the binary encoded instance *a*^(*b*)^ on *k* qubits. This operation was performed by employing the CNOT (i.e., controlled NOT gate) controlled on |c_1_⟩ = 0, which targeted the qubits correlating to the bit that is equal to one of the (*b* + 1)*^th^* instance *a*^(*b*)^.
- Reverse qubit $|c_{0}\rangle \;$ supposing the state of $|c_{1}\rangle =|0\rangle$. Also, apply the controlled rotation gate CG as defined below:
  (1) $${CG}_{\alpha }=\left[\begin{array}{cccc} 1 & 0 & 0 & 0\\ 0 & 1 & 0 & 0\\ 0 & 0 & \sqrt{\frac{\alpha -1}{\alpha }} & \frac{-1}{\sqrt{\alpha }}\\ 0 & 0 & \frac{1}{\sqrt{\alpha }} & \sqrt{\frac{\alpha -1}{\alpha }} \end{array}\right]$$
  Here, in Equation (1), α = G − *b* denotes the count of instances, along with the present one, which is yet to be determined. The gate $|c_{0}\rangle$ acts as control qubits and $|c_{1}\rangle$ acts as target qubits, respectively. This partition of qubits is intended to divide the quantum state across two branches *d*_1_ and *d*_2_. Branch *d*_1_ included primary (*b* + 1) occurrences in superposition, each of which had an amplitude of 1G, while the branch *d*_2_ included new occurrences $a^{\left(b\right)}$, with an amplitude of $\sqrt{1-b+1/G}.$ Here, branch *d*_1_ perpetually encoded binary occurrences until (*b* + 1)^th^.
- Finally, branch *d*_2_ is restored such that $|a^{\left(b\right)}\rangle ={|0\rangle }^{⨂k}$.
- Repeat from Step 1, to load subsequent instances to be encoded.

**Figure 2:**
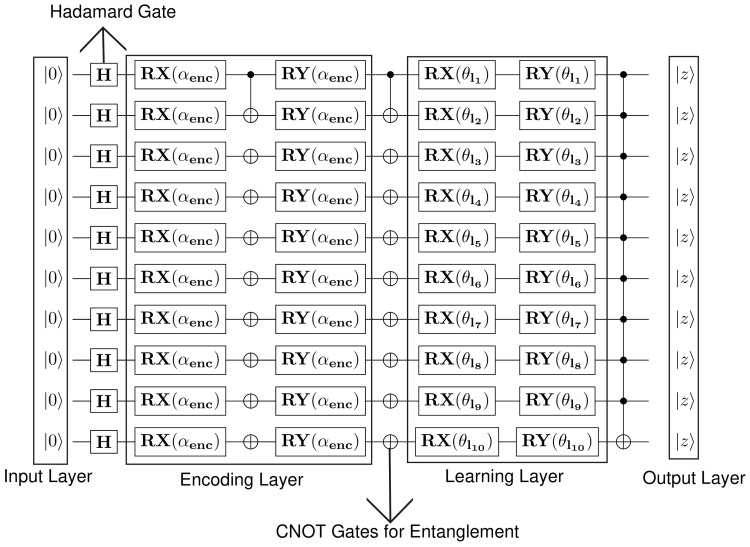
Quantum circuit designed for smart contract features.

The quantum circuit designed is represented in Figure 2. The circuit includes four layers, namely, input, encoding, learning and output layer. Between the input and encoding layers is the Hadamard gate, which superpositions the input qubits to the superposition phase. Further, amongst the encoding and learning layer is the CNOT gates, which are known for tangling and untangling the states. It is known to flip the target qubit if the value at control qubit is |1⟩. Lastly, the output layer projects the anticipated outcome based on the input units and operations performed. After encoding all the G binary occurrences, the system would be in the following state:

(2) $$|S\rangle =\frac{1}{\sqrt{G}}\sum_{b=0}^{G-1}|a^{\left(b\right)}\rangle$$

Here, in Equation (2), the quantum state |S⟩ has the estimate $\frac{1}{\sqrt{G}}$, for each instance a^(b)^, or 0 in other cases. As the count of amplitudes 2^G^ may be larger than the instances of nonzero amplitudes, sparse amplitude trajectories could be a part of the basis encoding features.

### 3.6. Detecting Vulnerabilities Using QML Approaches

The smart contract dataset *S* with *F* features of *G* instances is subjected to distinguishing vulnerabilities using QML algorithms. It is a typical classification problem, where instance *g* of the dataset *S* is categorized as vulnerable or non-vulnerable. Primarily, the quantum state |*S*⟩ is obtained by encoding the data *S* as defined previously. Further, basis encoding of instance *j* is to be classified via a supplementary register of *k*-qubits. Therefore, |j⟩ = |j_0_ j_1_ …… j_k−1_⟩ is obtained. Further, the circuit designed in Figure 2 is utilized to detect liabilities. For achieving this task, the study employed four popular QML learners, namely, QNN, Variational Quantum Classifiers (VQCs), Quantum Support Vector Machines (QSVMs) and Quantum Random Forests (QRFs). Each of these approaches is briefly described below:

- *QNN*: As the name suggests, QNN is centered on the concepts of neural network (NN) grounded on the fundamentals of quantum mechanics. The model accelerates learning towards better computation. Here, qubits portray the brain neurons [86]. Neuron models engross the quantum states for interpreting information. |0⟩ and |1⟩ are the quantum states representing classical bit information. The qubit state |γ⟩ sustains coherent superposition of quantum states |0⟩ and |1⟩.

(3) $$|\gamma \rangle =\beta |0\rangle +\gamma |1\rangle$$

In Equation (3), β and γ are probable amplitude values, which are generally complex numbers. They satisfy the condition mentioned in Equation (4).

(4) $${\left|\beta \right|}^{2}+{\left|\gamma \right|}^{2}=1$$

The neuron state in Equation (3) is rewritten as Equation (5).

(5) $$f\left(\delta \right)=e^{i\delta }=cos\delta +i\;sin\delta$$

The output state of QNN is based on two parameters: threshold ξ and phase estimate represented in the form of weighted associations $\theta_{i}$.

Also, $f\left(\delta \right)$ is an alternative representation of the quantum state, where δ is the phase of the quantum state. Weights of neuron state $x_{i}$ is represented by $f\left(\theta_{i}\right)$, where $f\left(\delta \right)$ converts input estimate $x_{i}$ into some quantum state with phase value $\theta_{i}$. Here, $x_{i}$ is the state of *i^th^* neuron. By multiplying the input $x_{i}$ with weight $f\left(\theta_{i}\right)$, the neuron would rotate, grounded on rotation gates. Based on these parameters, the output state is mathematically represented as follows:

(6) $$v=\sum_{i}^{I}f\left(\theta_{i}\right)\cdot x_{i}-f\left(\xi \right)=\sum_{i}^{I}f\left(\theta_{i}\right)\cdot \;f\left(y_{i}\right)-f\left(\xi \right),$$

(7) $$y=\frac{\pi }{2}\cdot g\left(\mu \right)-arg\left(v\right)$$

(8) H=f(y)

The state *v* of neuron is expressed as the weighted summation of input states subtracted from the threshold, as represented in Equation (6). The output qubit state *y* is adjusted roughly in Equation (7) as compared to Equation (6). Here, $arg\left(v\right)$ depicts the argument of v, which is a complex number. It is applied as arctanImvRev. Neuron state *H* is the output state of the QNN model. It is calculated as the weighted estimation of *i* discrete neuron states. Further, the activation function $g\left(\mu \right)$ used in Equation (7) deduces the general inverted illustration of quantum logic gates like CNOT. The value of *g* is computed using the sigmoid function:

(9) $$g\left(\mu \right)=\frac{1}{1+e^{-\mu }}$$

ii. *QSVM*: Using a kernel for quantum computing is the basis of QSVM. QSVM is the quantum variant of the classical SVM algorithm used in multiple ML tasks [87]. The quantum kernel is often estimated as an inner product of quantum states derived from two data coordinates. It can be mathematically defined as follows:

(10) $$K\left(t,\;t^{\prime }\right)={\left|\langle \emptyset \left(t\right)|\emptyset \left(t^{\prime }\right)\rangle \right|}^{2}={\left|\langle 0^{⨂m}|U^{t}U^{t^{\prime }}|0^{⨂m}\rangle \right|}^{2}$$

Here, two data points are considered as *t*, and *t′*. The kernel depicts the extent of similarity amongst two data points in high dimension space. The quantum kernel estimated using quantum circuit is displayed in Figure 2.

Further, for five instances of vulnerabilities in our dataset, the multiclass QSVM model was designed based on reducing the energy estimate derived from the quantum annealing approach to maximize the hyperplane [88]. Primarily, the SVM model is trained in an equivalent manner using quadratic programming (QP).

(11) $$\mathrm{minimize}\;\;\;\;\;\;\;\;\;\;\;\;\;\;\;\;\;\;E=\frac{1}{2}\sum_{m}^{G}\sum_{n}^{G}\alpha_{m}\alpha_{n}t_{m}t_{n}k\left(x_{m},\;x_{n}\right)-\sum_{m}\alpha_{m}$$

(12) $$\mathrm{such}\;\mathrm{that}\;\;\;\;\;\;\;\;\;\;\;\;\;\;\;\;\;\;\;\;\;\;\;\;\;\;\;\;\;0\leq \alpha_{m}\leq C$$

(13) $$\mathrm{and}\;\;\;\;\;\;\;\;\;\;\;\;\;\;\;\;\;\;\sum_{m}\alpha_{m}t_{m}=0$$

Here, in Equation (11), *G* is the number of instances of features in datasets and x_m_ ∈ $R^{e}$ is an instance in the *e*-dimensional feature space. Also, t_m_ = ±1 is the output label allocated to x_m_·α_m_ and ∈ R are the members of set with M coefficients, where *C* is the regularization estimate, and the kernel function is $k\;\left(\cdot \;,\;\cdot \right).$ The coordinates α_m_ are computed by SVM. It defines a (*e* − 1) dimension hyperplane, which partitions $R^{e}$ into two regions based on the class label. The kernel function used here is the Gaussian kernel.

(14) $$\alpha_{m}=\sum_{l=0}^{L-1}B^{l}q_{Am+l}$$

Here, in Equation (14), $q_{Am+l}\in \;\left\{0,\;1\right\}$ are binary estimates derived from the quantum annealer. *L* is the count of binary features incorporated into encode $\alpha_{m}$, while *B* is the base of encoding. Equation (11) is further modified as follows:

(15) $$E=\frac{1}{2}\sum_{m,n,l,o}q_{Am+l}q_{Am+o}B^{l+o}t_{m}t_{n}\cdot k\left(x_{m},\;x_{n}\right)-\;\sum_{m,l}B^{l}q_{Am+l}+\vartheta {\left(\sum_{m,l}B^{l}q_{Am+l}t_{m}\right)}^{2}$$

(16) $$E=\sum_{m,n=0}^{M-1}\sum_{l,o=0}^{L-1}q_{Am+l}{\tilde{Q}}_{Lm+o,Ln+o,\;Qn+o}$$

Here, in Equation (15), ϑ is the penalty metric, which is squared, and $\tilde{Q}$ is the LM × LM matrix. Employing the Karush–Kuhn–Tucker (KKT) constraints results in minimizing the energy metric using the Lagrangian function as follows:

(17) $$maximize\;\;\;\;\;\;\;\;\;\;\;\;\;\;\;\;\;\;\;\;\;\;\;\;\;\;\;\;\;W\left(\alpha \right)=\sum_{p=1}^{n}\alpha_{p}-\;\frac{1}{2}\sum_{p,\;q=1}^{n}y_{p}y_{q}\alpha_{p}\alpha_{q}\langle x_{p},y_{p}\rangle$$

$$such\;that\;\;\;\;\;\;\;\;\;\;\;\;\;\;\;\;\;\;\;\;\;\;\;\;\;\;\;\;\;0\leq \alpha_{p}\leq C,\;p=1,\;2,\;\cdots ,\;n$$

$$and\;\;\;\;\;\;\;\;\;\;\;\;\;\;\;\;\;\;\;\;\;\;\;\;\;\;\;\;\;\sum_{p=1}^{n}\alpha_{p}y_{p}=0$$

In Equation (17), W(α) is a function of α, whereas 〈x_p_, y_p_〉 is the kernel estimate of the Gaussian (or radial basis function, i.e., RBF) kernel. On solving these equations, QSVM estimates the largest margin amongst classes by minimizing the energy estimate. Thus, quantum annealer can be utilized to apply the QSVM model on multiclass problems.

iii. *VQC*: VQC is a variant of QNN employed for supervised learning. It optimizes the quantum circuit and minimizes the loss metric represented in triplet notation [89]:

(18) $$f\left(b,\;w,x\right)=y$$

In Equation (18), the network is represented by *f*, where the output neuron is *y*, which is used to compute the loss function L. Here, the network comprises three trainable parameters, i.e., *b*, *w* and *x*, the input data. Like QNN, the quantum circuit is modelled using CNOT and rotation gate. The gate rotates by learning from parameters γ, δ and ε, which are subsets of the weight matrix $W\;\in \;R^{q\times 3\times r}$, where *q* is the count of qubits and *r* is the number of layers in the VQN network. Each layer in the model includes two CNOT gates, a typical 2-qubit gate with no trainable metrics. Using a Hadamard date with a CNOT gate will establish entanglement in the circuit, which in turn repeals a qubic state based on the estimate of the alternative control bit. Once the gates are passed through the circuit, its output is estimated by employing the Pauli *Z* metric and deriving the expectation estimate *E*. Further, the model optimizes the estimates of *w* and *b* during the training process to derive the loss function. A forward pass is performed on the model, like a classical *NN* model, to estimate the loss function. Furthermore, backpropagation is performed to update the learnable parameters from network.

The mean square error (*MSE*) metric is computed for figuring the loss function, L.

(19) $$L=\frac{1}{r}\sum_{j=1}^{r}{\left(y_{j}^{real}-f\left(b,\;w,x_{j}\right)\right)}^{2}$$

It is the difference between real and predicted estimates. Further, the model will be trained, and backpropagation approaches are applied on the network by optimizing the parameters $\rho =\left(b,\;w\right)$. Fluctuations in output are captured by varying ρ as the gradient $\frac{\partial }{\partial \rho }\;f$. The gradient of network is estimated for quantum circuits using parameter-shift regulations.

- *QRF*: RF is a variant of the decision tree (DT) algorithm used for regression and classification tasks [90,91]. RF is composed of U discrete quantum DTs (QDTs), which form a group of weak ensembles, which is input for QRF. Given training data with *V* instances, $x_{v}^{train}\;\in \;R^{D}$, with class labels v_c_, $y_{v}^{train}=\left\{0,\;1,\;\cdots ,\;v_{c}-1\right\}$, to be trained from interpreted data, and ${W=\left\{\left(x_{v}^{train},y_{v}^{train}\right)\right\}}_{v=1}^{V}$, from underlying distribution E. Every tree is trained using the partition size (i.e., *V_p_* ≤ *V*). These instances are sampled from data *V*. When every classifier is trained, the model is evaluated on a separate dataset *V_train_,* by linking the distribution of forecasted class labels. The overall predictions are derived by averaging predictions from every QDT, which in turn yields a probabilistic distribution.

(20) $$Q_{r}\left(\vec{x};c\right)=Prob\left(c|\vec{x}\right)\;\forall \;c\in C$$

The predictions from QRF for an input $\vec{x\;}$ will be of the following form:

(21) $$\tilde{y}\left(\vec{x}\right)={argmax}_{c\;\in C}\left\{\frac{1}{T}\sum_{t=1}^{T}Q_{r}\left(\vec{x};c\right)\right\}$$

Accuracy of the model can be deduced by associating estimates from real instances. Probabilities of model overfitting are avoided by identifying differences amongst train and test datasets.

### 3.7. Architecture of QML Models

All QML models in this study used *PCA*-related feature vectors as input, followed by appropriate quantum feature encoding schemes. The architecture of each model is described below:

- *QNN*: The model is implemented as a layered variational circuit with *L* blocks. Each block comprises a data encoding layer, parameterized rotation layer and entangling layer. The unitary block l is given by
  (22) $$U^{\left(l\right)}\left(\theta^{l},\emptyset^{l}\right)={CNOT}_{ring}\cdot {⊗}_{i}R_{Y}\left(\theta_{I}^{\left(l\right)}\right)R_{Z}\left(\emptyset_{i}^{\left(l\right)}\right)$$
  The expected value $\langle Z_{0}\rangle$ of the first qubit was estimated and mapped to probability via $p=\left(1+\langle Z_{0}\rangle \right)/2$. The network was trained using the parameter-shift rule and Adam optimizer.
- *QSVM*: The model utilized an entangling feature map $U_{\emptyset }\left(x\right)$, where each qubit undergoes rotation operation $R_{Z}\left(\alpha_{i}x_{i}\right)$ followed by CNOT entanglement. The quantum kernel between two feature vector *x* and *x’* is computed using the SWAP test:
  (23) $$K\left(x,\;x^{\prime }\right){\left\lceil \langle 0|U_{\emptyset }{\left(x\right)}^{t}U_{\emptyset }\left(x^{\prime }\right)|0\rangle \right\rceil }^{2}$$
  The resulting kernel matrix is passed to a classical *SVM* solver with a precomputed kernel.
- *VQC*: VQC employs angle encoding followed by a strongly entangling ansatz. Each layer of the model comprises a rotation block and entanglement block. The output obtained from expectation values of designated qubits would be mapped to class probabilities of susceptibility, where cross-entropy was utilized for training.
- *QRF*: The ensemble of Quantum Decision Tree (QDT) is QRF. Each QDT encodes a random subset of features using *RY* rotations, followed by applying a shallow entangling circuit, and measures a decision qubit. Further, predictions from *T QDTs* are aggregated using the majority voting approach. Also, random feature selection and bootstrap sampling were applied to enhance diversity among trees.

## 4. Experiments

To ensure fair evaluation of diverse approaches, the models were trained and evaluated on homogeneous environments. All computations were accomplished on desktop with Intel I7 14700 K CPU clocked at 2.5 Ghz base frequency and 5.6 Ghz turbo frequency. We used RTX 3090 GPU with 24 GB VRAM, 32 GB RAM, deployed on Windows OS, with PyCharm software with version 2025.1.1, and python computing including Qiskit, Pennylane, TensorFlow dependencies being installed. An instance of smart contracts generated from expert audits is displayed in Table 3.

### 4.1. Hyperparameter Settings

To train the QML models on smart contract datasets, fine tuning of hyperparameters was accomplished. For the QNN model, Adam’s optimizer was employed to conduct a grid search for estimating optimal settings of the parameters. The search ranges for hyperparameters were as follows: batch size—(32, 64, 128); learning rate—(5 × 10^−5^, 1 × 10^−5^, 1 × 10^−4^, 4 × 10^−4^); completely connected neural layers—(1, 2, 3); dropout estimate—(0.1, 0.2. 0.3. 0.4. 0.5, 0.6); decay for learning rate, gamma—(0.97, 0.98, 0.99); L2 regularization—(1 × 10^−3^, 1 × 10^−4^, 1 × 10^−5^, 1 × 10^−6^); epoch count—(100, 200, 300, 400, 500). The loss was evaluated using the cross-entropy approach. The best estimates of parameters for the QNN model are enlisted in Table 4.

Similarly, ideal parameter settings for the QSVM model are represented in Table 5.

Optimal parameters for VQC are represented in Table 6.

Finally, parameters for the QRF approach are enlisted in Table 7.

### 4.2. Metrics for Performance Evaluation

The QML model’s performances were evaluated using conventional statistical approaches like precision, F1 score, accuracy and recall. These metrics are mathematically defined below:

- Precision, also known as positive predicted instances, estimates the ratio of accurately forecasted positive instances (i.e., true positives) to total count of instances predicted positive.

(24) $$Precision=\frac{True\;positives}{False\;positives+True\;positives}$$

- Accuracy indicates the inclusive amount of perfection in a mathematical model. It is the ratio of correctly predicted values (i.e., True negative, True positive) to the total count of data instances.

(25) $$Accuracy=\frac{True\;negatives+True\;positives}{True\;negatives+True\;positives+False\;negatives+False\;positives}$$

- Recall estimates the capability of a mathematical model to ascertain all positive occurrences. It is also called sensitivity and estimated as the ratio of accurately forecasted positive occurrences to all positive occurrences in data.

(26) $$Recall=\frac{True\;positives}{False\;negatives+True\;positives}$$

- F1 score is a significant estimate that combines recall and precision metrics. It finds out the balance and trade-off their estimates. The estimate is essential when the dataset is unbalanced.

(27) $$F1\;score=2\times \frac{Recall\times Precision}{Recall+Precision}$$

## 5. Results and Discussion

Here, SolidiFI data were utilized as test data and performance QML models were assessed as represented in Table 8. Each of these models were evaluated using precision, recall, accuracy and F1 score.

It is evident from the table that the performance of the QNN model is superior compared to other QML approaches. It has the maximum estimate for all statistical metrics. The model has the highest F1 score of 81.20%, which is better than the other models. It has learnt nonlinear decision boundaries through trainable rotation gates and entangling operations.

Additionally, the outcomes of performance estimates from five types of susceptibilities are reflected in Table 9.

From the above table, it is evident that the QNN model identifies all five vulnerabilities with better detection. It is also identified access control and reentrancy with better accuracy of 82.80% and 82.43%. The QNN model has better precision, accuracy, recall and F1 score as compared to other approaches in discovering liabilities. QNN, a parameterized quantum circuit, learns nonlinear boundary towards susceptible and non-susceptible smart contracts. Despite having the same training dataset, the performance of QML algorithms varies due to the diverse capabilities and generalizations of models. To conclude, the QNN model was identified as the best model in identifying susceptibilities in smart contracts.

Also, diverse tools were compared to understand their performance capabilities in detecting smart contract susceptibilities. The outcomes from comparative analysis are reflected in Table 10.

**Figure 3:**
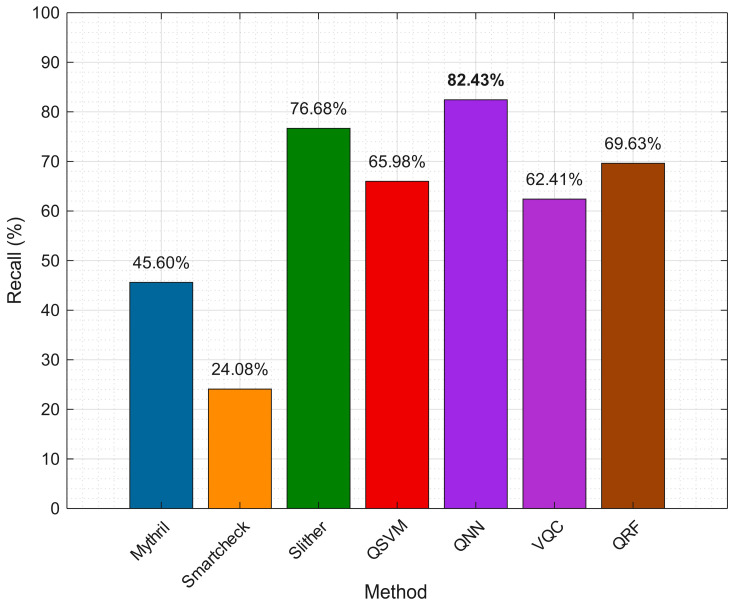
Recall metric for diverse smart contract vulnerability detectors.

The table portrays vulnerabilities detected from diverse tools and QML approaches. As tabulated, the QNN and Slither tool identified vulnerabilities better than other techniques. Smartcheck detected the least vulnerabilities from the dataset. These outcomes are further visualized in Figure 3 by comparing recall estimates from these detectors.

As the above figure depicts, the recall metric was compared with all the seven vulnerability detectors. Experimentations revealed that the QNN model had the highest recall rate of 82.43%, overriding Slither by 5.75%. The figure confirms the superior performance of the QNN model in discovering true positive occurrences of vulnerabilities. Other QML approaches like VQC, QSVM and QRF delivered lower recall values at 62.41%, 65.98% and 69.63%, respectively.

**Figure 4:**
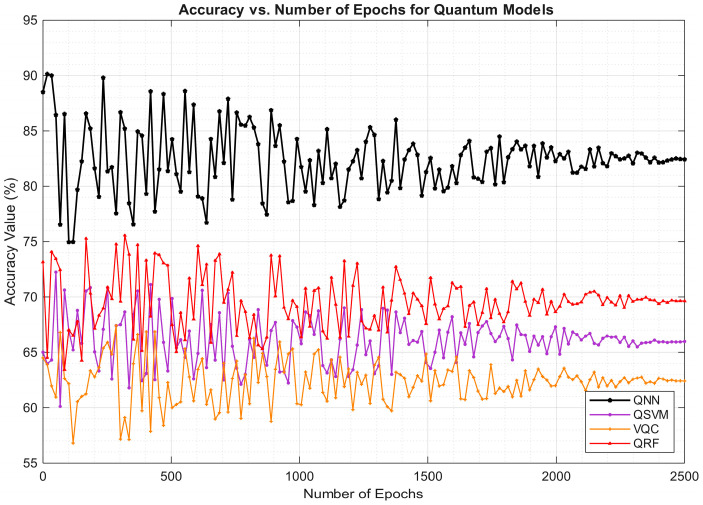
Accuracy analysis of QML approaches in detecting liabilities.

Also, performance accuracy was compared across QML approaches to analyze the capabilities of the algorithms. The analysis is visualized as Figure 4.

**Figure 5:**
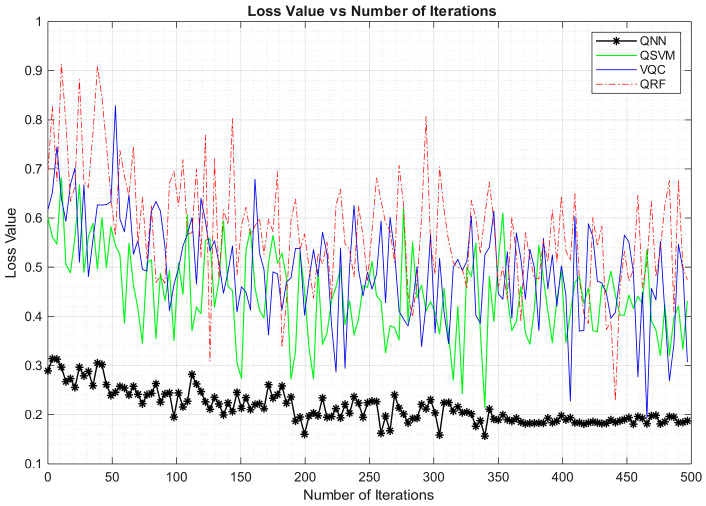
Loss function analysis of QML approaches in detecting vulnerabilities.

The figure reveals the superior performance of the QNN model with accuracy being stationary after 2000 epochs at 82.43%. On the contrary, the VQC algorithm achieves a lower accuracy of 62.41%. Similarly, loss function was evaluated and plotted for QML approaches to validate their performance. The visualization of loss function is depicted in Figure 5.

As observed from the figure, the conclusions are homologous with the accuracy analysis. At an epoch value of 400, the QNN algorithm’s loss function remains stationary with a minimal loss of 0.21. Other algorithms were not stabilized by 500 epochs with variations in loss function, as compared to the QNN model. To conclude, the QNN model achieves superior performance by achieving minimal loss value in deducing vulnerabilities.

### McNemar’s Test

Finally, McNemar’s test [92] was performed to further validate performances of the QNN approach in ascertaining liabilities in smart contracts. The consequences are tabulated in Table 11.

Table 10 depicts significant differences in accuracy with QNN and other QML approaches amongst four vulnerabilities, namely, gas limit, uninitialized storage pointer, access control and reentrancy. But there was very little difference in accuracy for overflow vulnerability. This might be due to the conspicuous features of overflow susceptibility, enabling the quantum model to identify and categorize it effectively.

Also, stability analysis was performed across ten different random seeds to validate the performance of the models. Under simulated depolarizing noise with *p* = 0.05 and *p* = 0.01, the QML models retained over 90% of noiseless accuracy, indicating robustness of hardware-level imperfections.

Both these tests validated the performance and generalization of models.

## 6. Conclusions

The present study employed QML approaches to detect vulnerabilities from smart contracts. Experimentations revealed that the QNN model outperformed other models in ascertaining susceptibilities. The models were assessed with statistical measures such as precision, accuracy and F1 metric. The study utilized three variants of smart contract data for training QML models. Initially, smart contracts were pre-processed to extract bytecodes from code segments. Further, bytecodes were encoded to a 258-dimention feature subset, which was further minimized to generate an optimal subset for quantum computing. Performances of all models were evaluated based on statistical metrics specific to each susceptibility. The QNN model was able to ascertain four types of vulnerabilities with minimal instances of false positives and false negatives. Other models were not superior in performance due to differences in training methodologies and generalization. In future, QML approaches can be employed in other areas of blockchain to minimize security threats and breaches [93,94].

Despite the promising outcomes, several shortcomings need to be addressed. For instance, all experiments were conducted on quantum simulators due to limited access to fault tolerant quantum hardware. Ral-device noise and decoherence effects were not completely accounted for. Also, the number of qubits available poses an upper bound on feature vector dimensionality, necessitating aggressive dimension reduction that may discard potentially relevant information. It is observed that these QML models performed on the benchmark smart contract dataset; therefore, their ability to generalize novel or emergent contract susceptibilities remains to be evaluated. In this direction, future work should focus on evaluating the proposed QML models on real quantum hardware, exploring hybrid quantum-classic architectures to minimize circuit depth while preserving accuracy and incorporating domain-specific feature engineering for smart contract analysis.

## Figures and Tables

**Table 1:** Notations of mathematical symbols used in study.

Symbol	Meaning	Dimension/Type
n	Number of smart contracts	Integer
m	Number of extracted opcode-level features	Integer
k	Number of principal components derived from PCA	Integer
X	Original feature matrix	$R^{n\times m}$
$\bar{x}$	Feature mean vector	$R^{m}$
X_c_	Mean-centered feature matrix	$R^{n\times m}$
Ʃ	Covariance matrix	$R^{m\times m}$
$\lambda_{i},w_{i}$	Eigenvalue, Eigenvector	$\mathrm{Scalar}/\;R^{m}$
W	PCA Projection matrix	$R^{m\times k}$
Z	Reduced feature matrix	$R^{n\times k}$
q	Number of qubits	Integer
θ	Trainable quantum circuit parameters	Real vector
${\hat{y}}_{i}$	Predicted output	Scalar
y_i_	True label	Binary
TP, TN, FP, FN	Confusion matrix terms	Integer

**Table 2:** Description of datasets employed for discovering vulnerabilities.

Data	Dataset	Instances	Susceptibilities Included				
			Gas Limit	Uninitialized Pointers	Access Control	Reentrancy	Overflow
Training	Slither dataset	10,000	Yes	Yes	Yes	Yes	Yes
	Smartbugs dataset	20,000	Yes	Yes	Yes	Yes	Yes
	Audited dataset	5000	Yes	Yes	Yes	Yes	Yes
Test	SolidiFI dataset	9369	No	Yes	No	Yes	Yes

**Table 3:** Sample smart contract dataset generated from expert audits.

Contract_ID	Bytecode	Susceptibility Identified
Contract_33	6080604052348015600e575f5ffd5b5060d580601a5f395ff3fe6080604052348015600e5..................................00081c0033	Normal
Contract_390	6080604052348015600e575f5ffd5b5060ba80601a5f395ff3fe6080604052348015600e57...........................................634300081c0033	Access control
Contract_959	6080604052348015600e575f5ffd5b506102268061001c5f395ff3fe60806040526004361061003....................................................4300081c0033	Reentrancy
Contract_2854	6080604052348015600e575f5ffd5b50610219861001c5f395ff3fe608060405234801561000f57.........................................................00081c0033	Gas limit
Contract_3750	6080604052348015600e575f5ffd5b506101218061001c5f395ff3fe6080604052348015600e57................................................634300081c0033	Overflow

**Table 4:** Optimized hyperparameters for QNN model.

Parameters	Optimal Configuration
Layers of network	3
Optimizer	Adam
Batch size	128
Learning rate	1 × 10^−5^
Dropout estimate	0.1
Decay of learning rate	0.97
L2 regularization	1 × 10^−3^
Loss metric	Cross entropy

**Table 5:** Optimized parameters for the QSVM model.

Parameters	Optimal Configuration
Regularization constant, C	0.8
No. of support vectors	14

**Table 6:** Optimized parameters for VQC model.

Parameters	Optimal Configuration
Layers of network	2
Quantum gate	CNOT
Batch size	64
Number of qubits	10
Optimizer	Adam
Loss metric	Cross entropy

**Table 7:** Optimized parameters for QRF model.

Parameters	Optimal Configuration
Number of decision trees	220
Maximum depth	9
Qubit count	20
Quantum gate	CNOT
Learning rate	1 × 10^−5^

**Table 8:** Comparison of QML algorithm performance.

QML Approach	Precision (%)	Accuracy (%)	Recall (%)	F1 Score (%)
QNN	83.50	82.43	81.17	81.20
QSVM	68.20	65.98	63.00	64.50
VQC	61.00	62.41	63.30	55.50
RF	70.10	69.63	63.50	60.20

**Table 9:** Comparison of performance of QML algorithms for smart contract liabilities.

Vulnerability	QML Model	Precision (%)	Accuracy (%)	Recall (%)
Gas limit	**QNN**	82.90	**81.55**	79.80
	QSVM	67.12	65.50	62.50
	VQC	60.50	61.12	61.80
	QRF	69.50	68.15	66.80
Uninitialized storage pointers	**QNN**	83.20	**82.10**	81.00
	QSVM	66.20	64.00	61.80
	VQC	62.50	61.90	60.00
	QRF	70.20	68.90	68.00
Access control	**QNN**	84.00	**82.80**	81.00
	QSVM	68.00	66.50	63.20
	VQC	62.50	61.90	60.00
	QRF	70.20	68.90	68.00
Reentrancy	**QNN**	83.50	**82.43**	81.50
	QSVM	67.50	66.00	63.00
	VQC	62.80	62.10	60.50
	QRF	70.50	69.20	68.20
Overflow	**QNN**	83.40	**82.25**	81.20
	QSVM	68.50	67.90	63.50
	VQC	63.00	62.41	60.20
	QRF	70.80	69.63	68.50

**Table 10:** Analysis of TP instances in detecting susceptibilities.

Vulnerability Detector	Gas Limit	Uninitialized Storage Pointers	Access Control	Reentrancy
Mythril	9	35	108	101
Smartcheck	0	28	92	82
Slither	35	66	198	300
QNN	65	89	225	351
QSVM	29	48	104	229
VQC	22	39	94	194
QRF	32	51	145	254

**Table 11:** Analysis of differences in accuracy amongst QNN and other QML models.

Vulnerabilities	Models	Discordant Pairs, i.e., B	Continuity Correction, i.e., C	Chi-Square Estimate	*p*-Value	Difference in Accuracy
Gas limit	QNN and other QML variants	3981	1	1873.92	0	Most significant
Uninitialized storage pointer		998	1	289.93	0	Most significant
Access control		783	1	209.62	0	Most significant
Reentrancy		2673	1	2341.89	0	Extremely significant
Overflow		3	1	0	1	Not significant

## Data Availability

Data will be made available on reasonable request.

## Abbreviations

The following abbreviations are used in this manuscript:

QML	Quantum Machine Learning
QNN	Quantum Neural Network
QSVM	Quantum Support Vector Machine
QRF	Quantum Random Forest
